# Modeling neurodevelopment in a dish with pluripotent stem cells

**DOI:** 10.1111/dgd.12699

**Published:** 2020-11-22

**Authors:** Kent Imaizumi, Hideyuki Okano

**Affiliations:** ^1^ Department of Physiology Keio University School of Medicine Tokyo Japan

**Keywords:** human‐specific neurodevelopment, neural specification, pluripotent stem cell, regional patterning

## Abstract

Pluripotent stem cells (PSCs) can differentiate into all cell types in the body, and their differentiation procedures recapitulate the developmental processes of embryogenesis. Focusing on neurodevelopment, we describe here the application of knowledge gained from embryology to the neural induction of PSCs. Furthermore, PSC‐based neural modeling provides novel insights into neurodevelopmental processes. In particular, human PSC cultures are a powerful tool for the study of human‐specific neurodevelopmental processes and could even enable the elucidation of the mechanisms of human brain evolution. We also discuss challenges and potential future directions in further improving PSC‐based neural modeling.

## Graphical abstract

In this review, we focus on pluripotent stem cell (PSC)‐based models for neural specification in early embryogenesis, patterning of the nervous system, and human‐specific neurodevelopmental process and discuss recent studies, remaining issues, and future strategies.

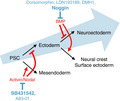

## 1 INTRODUCTION: THE ADVANTAGE OF PSC‐BASED MODELING IN NEURODEVELOPMENTAL BIOLOGY

Pluripotent stem cells (PSCs), including embryonic stem cells (ESCs) and induced PSCs (iPSCs), are characterized by the ability to both self‐renew and generate all cell lineages of the body (Jaenisch & Young, 2008). These PSC characteristics make it possible to generate any desired cell types in a culture dish. Indeed, many studies have demonstrated the directed differentiation of PSCs into various cell types by recapitulating the environments of embryogenesis. The differentiation of PSCs into neural cells has been reported for more than 25 years (Bain et al., 1995), and these technologies offer in vitro neurological disease models (Mattis & Svendsen, 2011) or cell sources for regenerative medicine (Okano & Sipp, 2020; Okano & Yamanaka, 2014). On the other hand, another important application of these technologies is the study of neurodevelopmental biology. Neurodevelopment is a complex process regulated by various factors, and while many studies on animal models, including *Drosophila*, *Xenopus*, chicks, and mice, have unveiled neurodevelopmental mechanisms, this complicated system has not been fully elucidated at the cellular or molecular level. PSC‐based modeling of neurodevelopment can provide a complementary system to in vivo animal models. PSC‐based modeling is less complex than animal models and lacks exogenous perturbations, and it is advantageous for specifying minimal requirements for developmental processes. In addition, the use of PSCs is especially useful for studying human‐specific neurodevelopment, which is difficult to investigate in animal models.

In this review, we focus on PSC‐based models for neural specification in early embryogenesis, patterning of the nervous system, and human‐specific neurodevelopmental process and discuss recent studies, remaining issues, and future strategies.

## 2 DIFFERENTIATION OF PSCS INTO THE NEURAL LINEAGE

**Figure 1:**
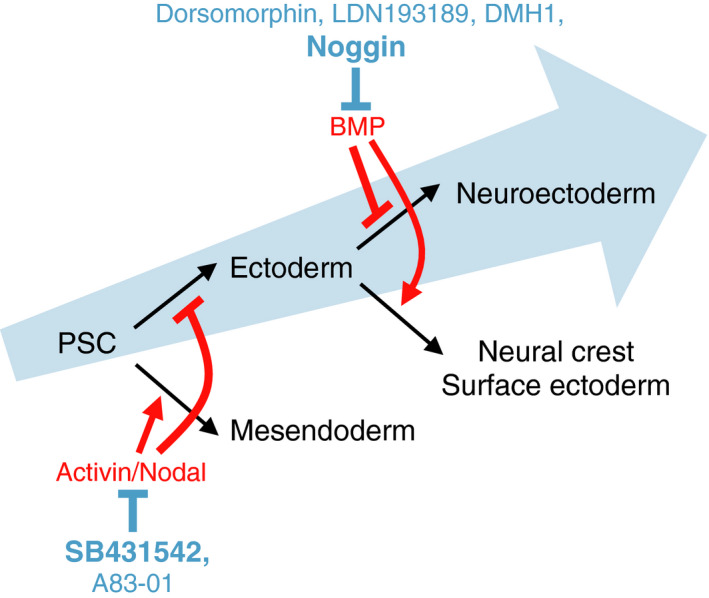
Directed differentiation of PSCs into the neural lineage by dual SMAD inhibition. Schematic for neural differentiation from PSCs. Dual SMAD inhibition leads to the selective induction of neuroectoderm by the prevention of non‐neural lineages. SB431542 and Noggin were initially used in the original report by Studer and colleagues, and other small‐molecule inhibitors targeting specific SMAD signaling pathways have now also been used

The initially reported method for neural induction from PSCs was the expansion of neural cells from randomly differentiated cell aggregations or embryoid bodies (EBs) (Bain et al., 1995; Reubinoff et al., 2001; Zhang et al., 2001). However, these methods are highly sensitive to variations from experiment to experiment. To overcome this issue, a concept called “dual‐SMAD inhibition (dSMADi)” was developed (Chambers et al., 2012), which enabled the directed induction of PSCs into the neural lineage. This concept relies on a large body of work in classical developmental biology. First, pioneering experiments of Spemann and Mangold (1924) demonstrated that the nervous system can be induced from the uncommitted ectoderm by signals that emanate from the dorsal lip of the amphibian blastopore (Spemann's organizer); then, experiments in *Xenopus* identified several molecules with neural inductive activity, including noggin (Lamb et al., 1993; Smith & Harland, 1992), follistatin (Hemmati‐Brivanlou et al., 1994), and chordin (Sasai et al., 1994). Later experiments demonstrated that these inductive molecules are antagonists for bone morphogenetic proteins (BMPs) and activin/nodal, both of which signal through key transduction components called SMADs (Muñoz‐Sanjuán & Brivanlou, 2002). Given that BMP activation leads to epidermal fate specification in the ectoderm (Wilson & Hemmati‐Brivanlou, 1995) and that activin/nodal promotes mesendodermal lineage induction (Asashima et al., 1990; Jones et al., 1995), these inhibitor molecules exert neural inductive activity via preventing the differentiation into non‐neural lineages. Based on these studies, Studer and colleagues developed the dSMADi method, in which PSCs were treated with the BMP inhibitor Noggin and the small‐molecule Activin/Nodal inhibitor SB431542, and rapid, highly efficient neural induction was accomplished (Chambers et al., 2012) (Figure 1). This method is now the gold standard for generating PSC‐derived neural cells. Of note, this study clearly demonstrated that dSMADi is the minimal requirement for neural specification in mammalian embryogenesis. As it is technically difficult to study the early stage of mammalian embryogenesis by using animal models, PSC‐based models can be powerful tools in this field. In addition to the neural lineage, the PSC‐based model has also indeed contributed to clarifying the molecular mechanism of early fate specification towards mesendoderm and non‐neural ectodermal lineages (Loh et al., 2014, 2016; Tchieu et al., 2017).

The study of PSC‐based neural induction models can also provide insights into the gene regulatory network of neurodevelopment. Apart from directed neural differentiation by dSMADi, Wernig and colleagues reported a novel neural induction method by forced expression of transcription factors in PSCs (Zhang et al., 2013). They demonstrated that *NEUROG2* overexpression was sufficient to activate the genetic program that drove neuronal differentiation. Direct neural differentiation from PSCs was also achieved by microRNA (miR‐9/9* and miR‐124) overexpression (Ishikawa et al., 2020). By using unbiased gene screening, some other master regulators of neurogenesis, such as *EZH2*, have also been identified (Wang et al., 2018; Nakatake et al., 2020). Such approaches would be helpful for clarifying the genetic programs orchestrating the neurodevelopmental process.

## 3 REGIONAL PATTERNING IN PSC‐BASED NEURAL MODELS

**Figure 2:**
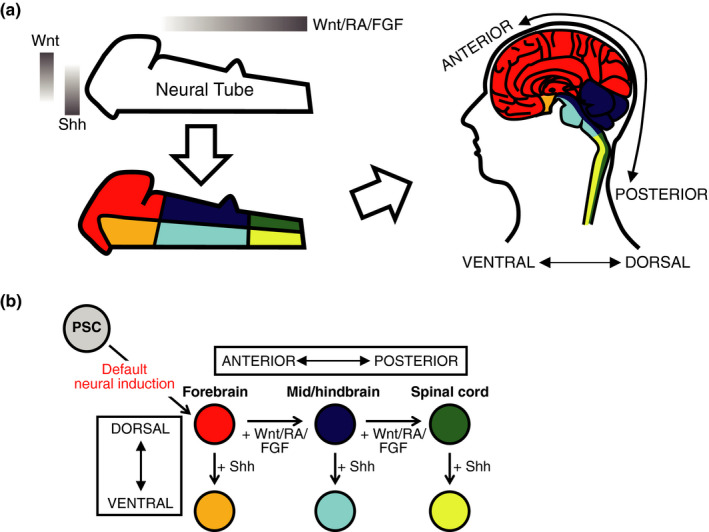
Regional control of PSC‐derived neural cells. (a) Signaling gradients along the anteroposterior (A‐P) and dorsoventral (D‐V) axes pattern the regional identity in the nervous system. (b) The control of A‐P and D‐V patterning signaling during neural induction enables the generation of any desired brain regions from PSCs

The developing nervous system is subdivided into distinct regions along the body axes, and each region produces a specific subtype of neurons. From the amphibian experiments, Nieuwkoop proposed a widely accepted two‐step model for this regional specification, known as “activation‐transformation” (Nieuwkoop et al., 1954; Stern, 2001). This model contends that ectodermal cells are first induced into a neural identity equivalent to the forebrain (“activation”), and cells subsequently enter into posterior fates, becoming midbrain, hindbrain, and spinal cord (“transformation”). In PSC‐based neural models, including EB‐based and dSMADi methods, differentiated cells default to forebrain specification (Chambers et al., 2012; Watanabe et al., 2005), demonstrating that the activation step in Nieuwkoop's model can be validated in PSC models. In addition, the PSC‐based neural model can also recapitulate the transformation step. In this step, transformation, or posteriorization, is modulated by various patterning signals, such as retinoic acid (RA) (Avantaggiato et al., 1996), Wnts (McGrew et al., 1995), and fibroblast growth factors (FGFs) (Cox & Hemmati‐Brivanlou, 1995; Kengaku & Okamoto, 1995). These signaling molecules are secreted from discrete organizers within or outside of the nervous system, and gradients of signaling molecules are established along the anteroposterior (A‐P) axis (Kiecker & Lumsden, 2012) (Figure 2a). PSC‐based neural models have demonstrated the in vitro recapitulation of this A‐P regionalization; for instance, treatment with moderate levels of RA endowed PSC‐derived forebrain‐type neural progenitors with midbrain/hindbrain identity, whereas high‐concentrations of RA converted these cells into spinal cord‐type neural progenitors (Okada et al., 2004, 2008). The posteriorization by canonical Wnt signaling was also demonstrated in PSC‐based models, in which distinct regionalized neural cells were obtained from PSCs (Kirkeby et al., 2012; Lu et al., 2015; Moya et al., 2014). Furthermore, a recent study artificially established a Wnt gradient in a culture dish by using microfluidic technology and mimicked A‐P patterning within PSC‐derived neural tissues (Rifes et al., 2020).

In addition to A‐P patterning, the nervous system is also patterned along the dorsoventral (D‐V) axis (Figure 2a). This D‐V patterning is mainly regulated by Wnt and Sonic Hedgehog (Shh) signaling. Wnt3a treatment led to the dorsalization of PSC‐derived neural cells, whereas Shh activation ventralized these cells (Okada et al., 2004, 2008; Watanabe et al., 2005). Notably, the combination control of A‐P and D‐V patterning enables the generation of any desired brain regions from PSCs (Imaizumi et al., 2015) (Figure 2b), and this technology is a powerful tool to model region‐specific disease phenotypes. For instance, iPSCs from patients with amyotrophic lateral sclerosis (ALS) exhibited abnormal neurite morphologies only when induced into spinal cord motor neurons but not into other brain regions, which is consistent with ALS pathology (Imaizumi et al., 2015). Such approaches will elucidate the mechanisms underlying selective vulnerability in neurological diseases (Fu et al., 2018).

Recently, a PSC‐based neural model has challenged Nieuwkoop's hypothesis of a basic theory for regional patterning. Briscoe and colleagues examined the dynamic change in chromatin accessibility during the induction from PSCs towards anterior (forebrain and hindbrain) and posterior (spinal cord) neural cells and revealed that cells commit to posterior identity before acquiring neural identity; that is, regional patterning (“transformation”) to some extent precedes neural specification (“activation”) (Metzis et al., 2018). The PSC‐based neural model not only recapitulates the neurodevelopmental process but also has great potential to redefine classical theories in neurodevelopmental biology.

## 4 HUMAN‐SPECIFIC DEVELOPMENTAL PROCESS IN PSC CULTURES

**Figure 3:**
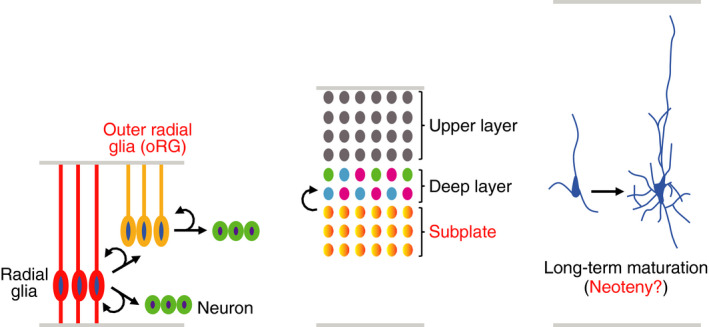
Characteristic neurodevelopmental processes in humans. Human PSC‐based neural models exhibit a prolonged period of progenitor expansion, the derivation of deep layer projection neurons from the subplate, and long‐term morphological/functional maturation, which may reflect the existence of outer radial glia (oRG) cells, the distinct regulation of deep layer neuron specification, and the human brain neoteny or retention of developmental traits into adulthood, respectively

The human nervous system is quite different from that of other species; however, the study of human neurodevelopment is an immature field because in vivo experiments of the human embryo are ethically challenging and technically difficult. In this sense, the human PSC‐based neural model can offer a complementary system. The cerebral cortex is one of the regions of greatest difference between humans and other species, and various groups have reported the derivation of cerebral cortex‐type neural cells from human PSCs. While these cells and mouse counterparts mostly share common features, including layer specificity (Espuny‐Camacho et al., 2013; Shi et al., 2012) and areal patterning (Imaizumi et al., 2018), human PSC‐derived cortical cells exhibit some distinct characteristics (Figure 3). One example is the prolonged period of progenitor expansion. In rodents, progenitor proliferation is followed by differentiation to neurons/glia, but human PSC‐derived cortical progenitors proliferate while also generating neurons (Otani et al., 2016). It seems that this protracted proliferation contributes to the size expansion of the human cortex. Notably, the developing human cortex contains an abundant population of specialized progenitors, known as outer radial glia (oRG) cells, which are not enriched in rodent brains (Hansen et al., 2010; Lui et al., 2011); thus, the unique characteristics of human PSC‐derived neural progenitors might mirror some aspects of oRG cells. In addition to progenitor expansion, some studies have implicated human‐specific regulation of neurons. Brivanlou and colleagues generated human PSC‐derived neurons with characteristics of the subplate, a postmitotic compartment that is disproportionately enlarged in humans (Ozair et al., 2018). They found that all major subclasses of deep layer projection neurons are mostly derived from subplate neurons and that the subclass specification within the subplate is regulated by Wnt signaling. This observation indicates that the regulation of cortical neuron specification is different between humans and rodents, which have relatively small subplates, resulting in complex neural circuits in the human brain. In addition, Vanderhaegen and colleagues demonstrated that, when grafted into the mouse brain, it took over 10 months for human PSC‐derived cortical neurons to reach the maturation stage with functional synapses and with electrophysiological activities (Linaro et al., 2019). This is in contrast to mouse neurons, which mature in only a few weeks. This may reflect the neoteny or retention of developmental traits into adulthood. Indeed, in the human prefrontal cortex, dendritic morphogenesis continues over 10 years of age, and synaptic reorganization extends into the third decade of life (Petanjek et al., 2011). A longer period of dendritic and synaptic organization would result in higher complexity and more numerous connections in the human brain.

It is also noteworthy that the abovementioned human‐specific characteristics observed in PSC‐based models are remarkable, even compared with non‐human primates (NHPs); for instance, human PSC‐derived neural progenitors have a much longer period of expansion than those of macaques (Otani et al., 2016), and the timing of neuronal morphological and functional development is longer in human PSC‐based models than it is in chimpanzee counterparts (Marchetto et al., 2019). Not only are there primate‐rodent differences but there are also differences between humans and NHPs that are considered to have a key role in human brain evolution. In fact, genomic analyses identified genes that newly emerged, duplicated, or changed their regulatory elements after human‐chimpanzee divergence, and rodent or NHP model experiments indicated the involvement of these genes in brain developmental processes (Table 1) (Charrier et al., 2012; Florio et al., 2015; Heide et al., 2020; Ju et al., 2016; McLean et al., 2011; Suzuki et al., 2018). However, it is not easy to demonstrate the significance of these genes in human brain development by using such animal models; thus, the human PSC‐based model is also a powerful system to approach human‐NHP differences. Indeed, some groups have reported differences in the gene expression regulation and chromatin accessibility in PSC‐derived neural cultures between humans and chimpanzees (Kanton et al., 2019; Pollen et al., 2019). These studies would contribute to understanding the molecular basis of human brain evolution, especially after divergence from the NHP lineage.

**Table 1:** Genes involved in human brain evolution

Gene	Change after the *Homo‐Pan* divergence	Consequence of genetic change	Effect on brain development	References
*SRGAP2C*	Gene duplication	Inhibiting ancestral SRGAP2A	Delaying spine maturation and increasing spine density	Charrier et al. (2012)
*ARHGAP11B*	Gene duplication	Gaining a new function	oRG expansion	Florio et al. (2015); Heide et al. (2020)
*TBC1D3*	Gene duplication	Increasing expression level	oRG expansion	Ju et al. (2016)
*NOTCH2NLB*	Gene duplication	Activating the Notch pathway	Increasing self‐renewal of cortical progenitors	Suzuki et al. (2018)
*GADD45G*	Enhancer loss	Brain‐specific loss of GADD45G expression	Evading a negative regulation of tissue proliferation by GADD45G	McLean et al. (2011)

## 5 CURRENT ISSUES AND FUTURE OUTLOOK FOR NEURAL MODELING BY PSCS

Although the PSC‐based model has succeeded in recapitulating some important aspects of neurodevelopment, there remain some issues for addressing neurodevelopmental processes by using PSC cultures. The first problem is the recapitulation of brain morphology. Typical 2D cultures cannot reproduce characteristic structures of developing brains, such as apicobasal polarity of the neuroepithelium and laminar formation of the cortical plate. Sasai and colleagues found a potential solution for this problem by generating 3D cultures of PSC aggregates by directing them into the neural lineage (Eiraku et al., 2008). In their culture, the aggregates underwent self‐organized formation into typical structures of cortical tissues. This 3D culture mimicking structural aspects of the developing brain is being improved by a variety of groups, and this type of culture is now widely accepted as a “brain organoid” (Kadoshima et al., 2013; Lancaster et al., 2013; Pasca et al., 2015; Qian et al., 2016). Much attention has been paid to this research field, combined with chromatin accessibility analysis and single‐cell transcriptomics (Trevino et al., 2020; Velasco et al., 2019).

Another difficulty, especially in human PSC cultures, is the long duration of neuronal maturity. It is true that the long period of morphological and functional maturation is characteristic of human brain development, but cumbersome long‐term culture is a practical obstacle. To overcome this problem, the overexpression of master regulator genes, such as *NEUROG2*, in PSCs can shortcut the neural differentiation processes, and electrophysiologically active neurons can be obtained in a few weeks (Y. Zhang et al., 2013). However, it is still controversial whether this rapid gene‐based neuronal induction from PSCs can fully recapitulate all the aspects of mature neurons, because some features of postnatal mature neurons are observed in native directed differentiation that occurs over 6 months, but not in the gene‐based differentiation method (Rosa et al., 2020).

An important future direction in PSC‐based neural modeling will be to thoroughly pursue approaches that are difficult to achieve in embryological animal experiments. One of the most prominent examples is interspecies comparisons. As mentioned above, some studies have focused on human‐rodent and human‐NHP differences. We envisage that the next step will be to establish a novel platform enabling parallel comparative analyses among various species and to comprehensively elucidate species‐general and species‐specific mechanisms of neurodevelopmental processes. Another example in this direction of research is the advancement of reconstitutive approaches. The in vitro reconstitution of neurodevelopmental processes, from neural specification to local brain morphogenesis, has been successful, as discussed above, to some extent. For tackling complex mechanisms in embryogenesis, however, a more global level of reconstitution is necessary, and that would include the interregional cross‐talk within the nervous system and the interactions between the nervous and non‐nervous systems. Indeed, recent studies recapitulated some aspects of the interbrain interactions by assembling region‐specific brain organoids (Bagley et al., 2017; Birey et al., 2017; Xiang et al., 2017), the juxtaposition of neural and non‐neural ectoderm in micropatterned cultures (Haremaki et al., 2019), and the interplay between neural cells and mesoderm‐derived microglia in organoids (Ormel et al., 2018). Thus, it may be feasible in the near future to realize the full reconstitution of embryogenic processes from the cell to organ scale.

## 6 CONCLUSIONS

Pluripotent stem cells cultures can recapitulate various stages of neurodevelopment, including neural specification and regional patterning. These recapitulation models are mainly based on a large body of work in embryological animal experiments, but PSC‐based models have also provided novel insights into neurodevelopmental processes. In particular, human PSC cultures are a powerful tool for studying human‐specific neurodevelopment and will contribute to the elucidation of the mechanisms of human brain evolution.

## CONFLICT OF INTEREST

H.O. is a compensated scientific consultant for San Bio Co. Ltd. and K Pharma Inc. K.I. declared no potential conflicts of interest.
